# Genetic alterations of *KRAS* and *TP53* in intrahepatic cholangiocarcinoma associated with poor prognosis

**DOI:** 10.1515/biol-2022-0652

**Published:** 2023-07-17

**Authors:** Jianbo Peng, Shuo Fang, Meisheng Li, Yuxin Liu, Xiaolu Liang, Zuobiao Li, Gaohui Chen, Lijiao Peng, Nianping Chen, Lei Liu, Xiaohong Xu, Wei Dai

**Affiliations:** Foshan Traditional Chinese Medicine Hospital, Guangdong, 518000, China; Department of Oncology, The Seventh Affiliated Hospital, Sun Yat-sen University, Shenzhen, Guangdong, 518000, China; Foshan First People’s Hospital, Guangdong, 518000, China; Guangdong Medical University, Zhanjiang, Guangdong, 524000, China; Department of Hepatobiliary and Pancreatic Surgery, Affiliated Hospital of Guangdong Medical University, Zhanjiang, Guangdong, 524000, China; Department of Oncology, Affiliated Hospital of Guangdong Medical University, Zhanjiang, Guangdong, 524000, China; Affiliated Hospital of Guangdong Medical University, Zhanjiang, Guangdong, 524000, China; Department of Ultrasound, Affiliated Hospital of Guangdong Medical University, Zhanjiang, Guangdong, 524000, China

**Keywords:** intrahepatic cholangiocarcinoma, next-generation sequencing, genetic profile, tumor mutation burden, prognosis analysis

## Abstract

The aim of this study is to investigate certain genetic features of intrahepatic cholangiocarcinoma (ICCA). A total of 12 eligible ICCA patients were enrolled, and tumor tissues from the patients were subjected to next-generation sequencing of a multi-genes panel. Tumor mutation burden (TMB), mutated genes, copy number variants (CNVs), and pathway enrichment analysis were performed. The median TMB was 2.76 Mutation/Mb (range, 0–36.62 Mutation/Mb) in ICCA patients. The top two most commonly mutated genes in ICCA were *KRAS* (33%) and *TP53* (25%). The co-mutations of *KRAS* and *TP53* were 16.7% (2/12) in ICCA patients. Notably, patient P6 with the highest TMB did not have *KRAS* and *TP53* mutations. Additionally, *TP53* and/or *KRAS* alterations were significantly associated with poor progression-free survival than those with wild type (1.4 months vs 18 months). DNA damage repair and homologs recombinant repair deficiencies were significantly associated with high TMB in ICCA cases. In conclusion, we found that certain genetic mutations of *TP53* and *KRAS* could predict poor prognosis in ICCA patients.

## 1 Introduction

Cholangiocarcinoma (CCA) is a malignant tumor of the liver originating from the cholangiocytes of the bile ducts [1,2]. According to its anatomical location, CCA is mainly classified according to the primary anatomic subtype as intrahepatic cholangiocarcinoma (ICCA), perihilar cholangiocarcinoma (pCCA), and distal cholangiocarcinoma (dCCA) [3,4]. To date, CCA has presented an increased incidence rate and unfavorable prognosis [1,2,5,6,7,8]. The estimated ICCA incidence increased in the majority of registered countries from 1993 to 2012, especially in Asia, including South Korea, Thailand, and China [6]. A prevalence investigation in the Chinese population reported that the incidence of ICCA increased significantly from 0.6 per 100,000 in 2000 to 1.3 per 100,000 in 2018. Additionally, in the past decade, the OS of ICCA has not improved significantly [1,2,8]; the median overall survival (OS) of the ICCA patients was 13 months, and 5-year OS rates were 13.79% [7], and tumor recurrence rates after resection are still disappointed [9]. At present, the survival rate of liver cancer is much lower in China than in developed countries [10].

The standard of clinical therapies for advanced CCA includes cisplatin or gemcitabine, but the response rate to these chemotherapies is poor, and consequently, they show poor prognosis with only 5–10% of 5-year survival [1]. Encouragingly, the immunogenomic traits of ICCA are intrinsically heterogeneous among patients, bringing both challenges and opportunities to personalized immunotherapy [11]. Depending on the specific genetic profile of each tumor sample, the combinatorial therapy of immunotherapy with traditional treatment and small molecular inhibitors shed light on personalized treatments [12,13,14]. Differences in the molecular profile between the subtypes of CCA have been presented in the frequency of mutations in certain genes, rather than different sets of genes being mutated [15]. *TP53* and *KRAS* seemed to be documented commonly both in CCA and other pan-cancers [15,16,17,18,19,20,21,22,23,24,25]. For instance, Guo et al.’s study showed that *TP53* and *KRAS* were the common high-frequency mutation genes in ICCA cohorts; more importantly, univariate and multivariate analyses discovered that *TP53* and *KRAS* mutations were associated with poor prognosis [17]. Through the investigation of mouse models with biliary tract cancer driven by *TP53* loss, the reprogramming of hepatocytes to cholangiocytes was strengthened to facilitate the formation of hepatocyte-derived ICCA. Moreover, ICCA driven by *KRAS* and *TP53* may originate from both mature cholangiocytes and hepatocytes [18]. Genetic alterations, including *TP53*, *BRCA1*, *BRCA2*, *BRAF,* and *KRAS*, had been demonstrated to be associated with cancer survival in 210,802 pan-cancer patients [25]. However, the comprehensive genetic features of ICCA remain to be investigated further and widely.

Our study aims to perform targeted next-generation sequencing (NGS) panels for comprehensively exploring the molecular characteristics of ICCA in a cohort of 12 ICCA patients. Furthermore, we also investigated the associations between clinical outcome and tumor mutation burden (TMB) or certain co-mutations of *TP53* and *KRAS*, respectively. Our findings predicted that ICCA harbored certain distinct genetic alterations were vulnerable to poor prognosis.

## 2 Materials and methods

### 2.1 Patients

Patients were recruited from January to December in 2020 and followed up for 18 months visit. All cases were histologically confirmed by two experienced pathologists. Written informed consent form was obtained from each patient in the Affiliated Hospital of Guangdong Medical University. The clinical data, OS, and progression-free survival (PFS) rates were collected. The TCGA-CHOL (The Cancer Genome Atlas) dataset was downloaded from the UCSC Xena (https://xenabrowser.net/datapages/), and the BTCA-JP dataset was downloaded from the ICGC database (International Cancer Genome Consortium) (https://dcc.icgc.org/).


**Informed consent:** Informed consent has been obtained from all individuals included in this study.
**Ethical approval:** The research related to human use has been complied with all the relevant national regulations and institutional policies and in accordance with the tenets of the Helsinki Declaration, and has been approved by the Ethics Committee of Affiliated Hospital of Guangdong Medical University (No. PJ2020-048).

### 2.2 DNA isolation and purification

Genomic DNA was extracted from formalin-fixed paraffin-embedded (FFPE) samples using the Tissue Kit (QIAGEN, Venlo, Netherlands). The quality of purified DNA was assayed by gel electrophoresis and quantified by the Qubit^®^ 4.0 fluorometer (Life Technologies, USA).

### 2.3 NGS and bioinformatics analysis

The targeted libraries were constructed using NGS Fast DNA Library Prep Set (Thermo Fisher, Waltham, MA, USA). The quality of the obtained libraries was evaluated by Agilent2100 Bioanalyzer (Agilent Technologies). ZhenXinan ctDNA NGS Panel (Tongshu BioTech, Shanghai, China) targeted NGS including 556 genes was performed on Ion Torrent (Tongshu BioTech, Shanghai, China) [26]. BWA (BurrowsWheeler-Alignment) software was used to compare the sequencing results. GATK (The Genome Analysis Toolkit) was used to correct the comparison quality. R package maftools (v2.4.15) software was used to detect somatic mutation, and an oncoplot map was drawn. Non-synonymous somatic mutations, including missense, nonsense, splice-site, inframe, and frameshift mutations, were included in our analyses. To calculate the TMB per megabase, the total number of mutations counted is divided by the size of the coding region of the targeted territory [27]. Copy number variation analysis was conducted using CNVkit (v0.9.6) and gistic2 (v2.0.23). Assignment of +2 or −2 of DNA copy number was considered the cut-off for amplification or deep deletion, respectively [28]. Gene rearrangement analysis was discovered using factera v1.4.4 [29]. Gene Ontology (GO) and Kyoto Encyclopedia of Genes and Genomes (KEGG) functional enrichment analysis was conducted using clusterProfiler (v3.14.3) function of R and visualized by ggplot2 (v3.3.6) [30].

### 2.4 Statistical analysis

All analyses used R (V.3.6.1) packages. The chi-square test, Fisher’s exact test, Student’s *t*-test, and Wilcoxon or Mann–Whitney test were used for intergroup comparison as needed. Two-tailed tests were used, and *p* < 0.05 was considered statistically significant. Survival curves were plotted using the Kaplan–Meier method and analyzed by survival (v3.3) and survminer (v0.4.9). As long as the patient contains the mutated genes on the specified pathway, this patient was divided into the mutant-type group, otherwise was divided into the wild-type group. *P* values of pathways with prognosis between these two groups were then calculated according to the PFS/OS time (the Wilcoxon test). Gene signatures of mutations were screened using univariate Cox models. Samples were classified as DDR positive or DDR negative depending on whether they contained any DDR (DNA damage response and repair) genes.

## 3 Results

### 3.1 Patient characteristics

A total of 12 patients with ICCA were enrolled in this study, including 8 males and 4 females (Table 1). As shown in Table 1, the median age of patients with ICCA was 64 years old (range, 40–78 years), seven (58.3%) with ECOG score 0 and two (16.7%) with score 2, and the rest four with the unknown score. The median tumor size was 180 cm^3^ (range, 4–1765.8 cm^3^), and five patients with smaller tumor volumes (<180 cm^3^) were all exclusive females. At the time of initial diagnosis, 41.7% (5/12) of patients were at stages I and II, while 58.3% (7/12) were at stages III and IV. For metastasis, 66.7% (8/12) of the patients were involved in at least one organ metastasis, including distal lymph node, liver, and peritoneum. Interestingly, primary ICCAs were located mostly (9/12) at the right liver. There were eight ICCA patients who undergone immune checkpoint inhibitors (ICIs) (first and second lines). The median OS and PFS rates were 18 months (range, 2.5–18 months) and 3.5 months (range, 0.6–18 months), respectively. All 12 patients were examined to be microsatellite stable (MSS), and only 2 of them were PD-L1 positive.

**Table 1:** Demographic information of patients with ICCA

	ICCA (*n* = 12)
**Age at initial diagnosis (years)**	
Median (range)	64 (40–78)
**Gender**	
Male	8 (66.7%)
Female	4 (33.3%)
**ECOG PS at initial diagnosis**	
0–1	7 (58.3%)
2–3	2 (16.7%)
Unknown	3 (25%)
**Tumor size (cm** ^**3**^)	
Median (range)	180 (4–1765.8)
**Stage**	
Stage I	2
Stage II	3
Stage III	6
Stage IV	1
**Metastasis**	
None	4
Liver, lymphoid, gallbladder, peritoneum	8
**Primary site**	
Left	3
Right	9
**MSS**	12
**PD-L1**	
Positive	2
Negative	10
**Immunotherapy**	
First line	4
Second line	4
None	4
**PFS median (range)**	3.5 (0.6–18)
≥Median	6
<Median	6
**OS median (range)**	18 (2.5–18)
≥Median	8
<Median	4

### 3.2 Mutation frequency and significantly mutated genes between ICCA and matched control pairs

**Figure 1:**
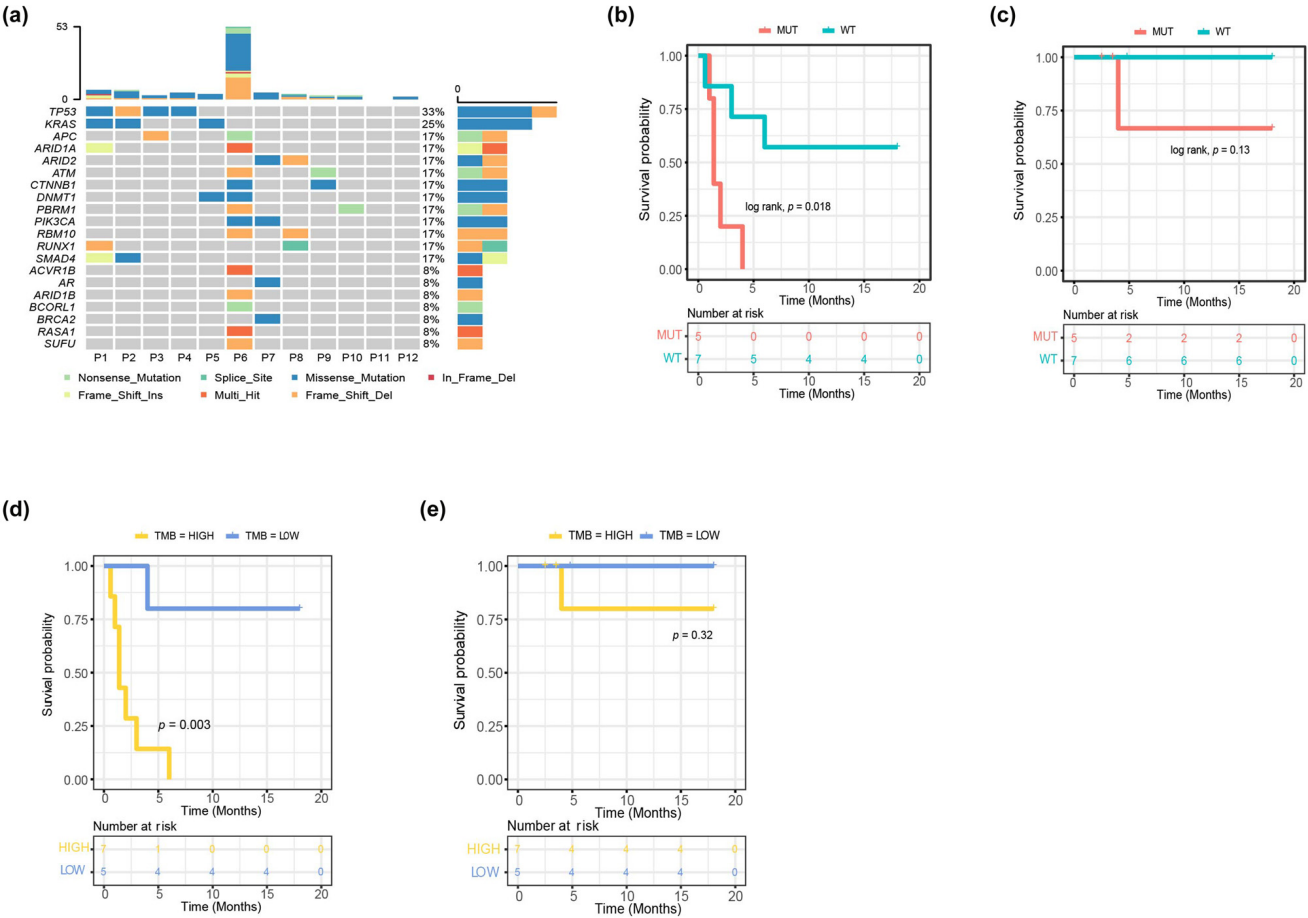
Genomic characteristics of ICCA. (a) Waterfall plots showing the frequency and types of mutations found in the TOP20 mutated genes in ICCA; (b) PFS and (c) OS of all patients stratified by TP53 and or KRAS mutation versus wild type (MUT vs WT). (d) PFS and (e) OS of all patients stratified by TMB high and TMB low.

The genetic alterations of the ICCA cohort are shown in Figure 1. The top two most commonly mutated genes in ICCA were *KRAS* (33%) and *TP53* (25%) (Figure 1a). Among mutation subtypes of top 20 frequently mutated genes, such as missense, nonsense, shift, splice, and multi-hit, missense mutations were accountable for approximately 44.4% (16/36). Two patients had the co-mutations of *KRAS* and *TP53* (16.7%). Interestingly, patient P6 who had the most genetic alterations did not have *KRAS* and *TP53* mutations. On the contrary, patients P11 and P12 did not have any genetic mutations. Specifically, the patients who harbored any one of the *KRAS* and *TP53* mutations survived for a significantly shorter period of PFS than those with wild type (1.4 months vs 18 months, *p* = 0.018) (Figure 1b). However, there were no significant associations with OS between the patients with *KRAS* and/or *TP53* mutations and those without *KRAS* and *TP53* mutations (4 months vs 18 months, *p* = 0.13) (Figure 1c). We further validated the association between these two genes and prognosis in the cohort of ICCA from the ICGC database. In the ICCA patients from the ICGC database, the *TP53* and/or *KRAS* mutations were significantly associated with PFS and OS (*p* < 0.0001 and *p* = 0.00034, respectively) (Figure S1). In addition, we investigated the survival rates of both PFS and OS in ICCA patients with *KRAS* and/or *TP53* mutations who received ICI treatment. However, no significant difference was observed in the survival rates of patients who received ICI therapy (Table S1).

After calculating, it was found that the median number of tumor mutations burden was 2.76 Mutations/Mb, ranging from 0 to 36.62 Mutations/Mb. Patients with high TMB (≥2.76 Mutations/Mb) had a worse PFS than those with lower TMB (<2.76 Mutations/Mb), with a PFS of 1.4 vs 18 months (*p* = 0.003) (Figure 1d). However, no significant difference was detected in OS based on TMB (*p* = 0.32) (Figure 1e). Furthermore, TMB was not significantly associated with clinical features, including age, stages, metastatic organs, tumor volume, and tumor original sites, as well as patients with *KRAS* and *TP53* mutations (Table S2).

### 3.3 Genetic signature unavailable to predict prognosis

In the present study, it was initially anticipated that genetic signatures could be used to predict clinical outcomes. However, no significant difference was found after using the univariate Cox model to analyze the top 20 mutated genes that might impact the overall prognosis (Table S3). As a result, no genetic signature was identified that could be further analyzed using the multivariate Cox model or LASSO regression model.

### 3.4 CNV and gene rearrangement analysis

**Figure 2:**
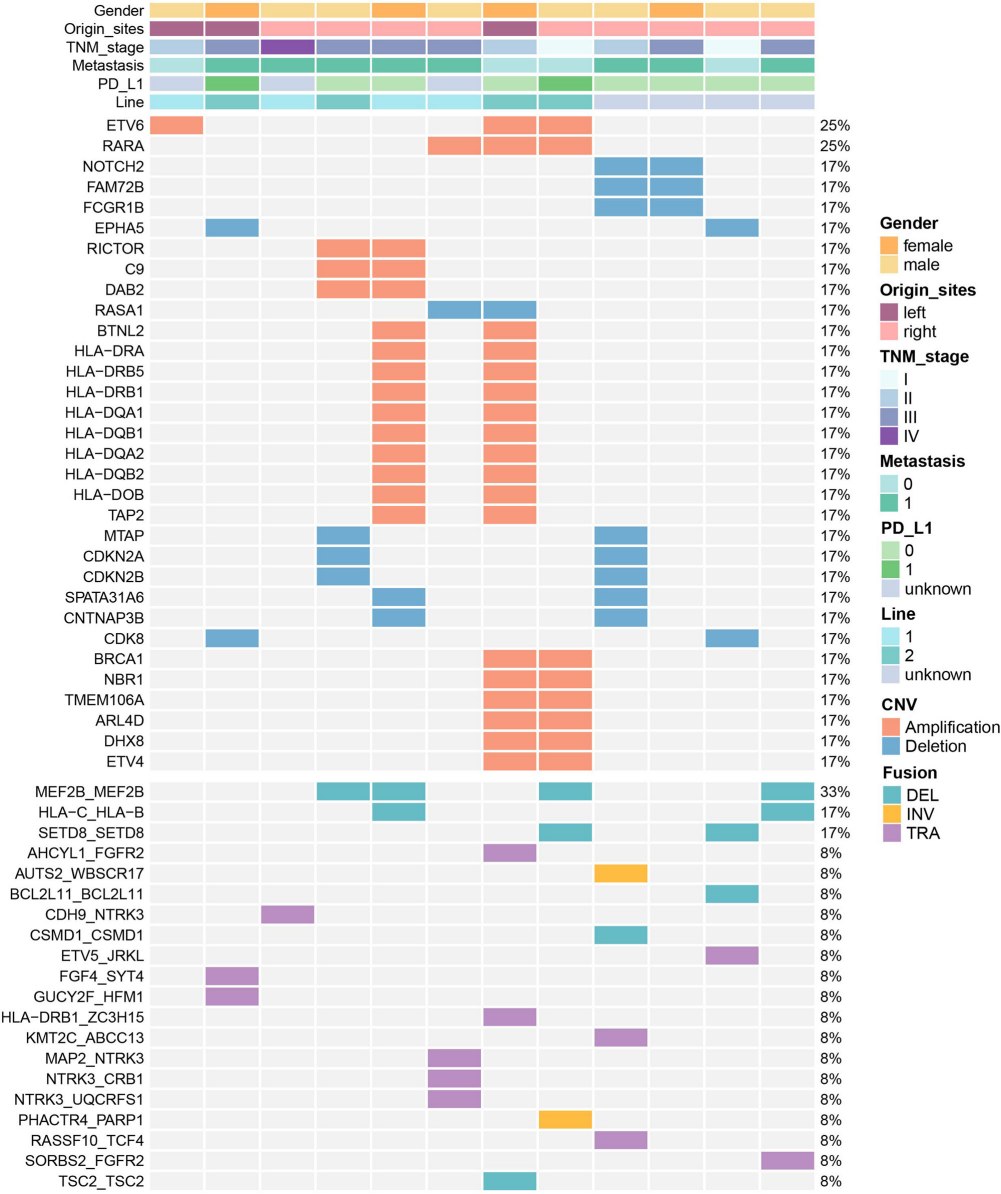
Distribution of copy number variation and gene fusion. The gender of patients was provided as bars on the top, followed by original site, TNM stage, metastasis, PD-L1, and immune checkpoint inhibitor (ICI) treatment (line1/2 means ICI drugs, unknown means no ICI treatment). The mutation types were indicated by the color on the right. Each column represents one patient.

Copy number differences are a common type of aberration, and in this study, we used CNVkit and GISTIC2 to detect focal changes in copy numbers (Figure 2 and Table 2). The results showed that *ETV6* and *RARA* were amplified in 25% of the samples (3/12). Furthermore, *RICTOR*, *C9*, *BRCA1*, and *HLA-DRA* were found in 16.7% of the samples (2/12), respectively. Similarly, *NOTCH2*, *FAM72B*, *CDKN2A*, *CDKN2B*, and *CDK8* were also observed in 16.7% of the samples (2/12). Rearrangements were mostly annotated in *MEF2B*_*MEF2B* fusion in 33.3% of the samples (4/12). As shown in Figure 2, female patients exhibited a higher frequency of CNVs than males, and the distribution of CNVs varied across other clinical features such as original site, TNM stage, PD-L1 expression, and metastatic status. However, for gene rearrangement, samples with higher CNV mutations contain 2–4 gene fusions, including translocation, inversion, and deletion (Figure 2 and Table 2).

**Table 2:** Frequent mutated genes with CNV and rearrangement

Gene	Type	Number (%)
**CNV**	**Amplification**	
	*ETV6*/*RARA*	3 (25%)
	*RICTOR*/*C9*/*HLA*-*DRA*/*BRCA1*	2 (16.7%)
	**Deletion**	
	*NOTCH2*/*FAM72B*/*CDKN2A*/*CDKN2B*/*CDK8*	2 (16.7%)
**Fusion**	**Translocation**	
	AHCYL1_FGFR2/CDH9_NTRK3/ETV5_JRKL/FGF4_SYT4/GUCY2F_HFM1/HLA-DRB1_ZC3H15/KMT2C_ABCC13/MAP2_NTRK3/NTRK3_CRB1/NTRK3_UQCRFS1/RASSF10_TCF4/SORBS2_FGFR2	1 (8.3%)
	**Inversion**	
	AUTS2_WBSCR17/PHACTR4_PARP1	1 (8.3%)
	**Deletion**	
	MEF2B_MEF2B	4 (33.3%)
	HLA-C_HLA-B/SETD8_SETD8	2 (16.7%)
	BCL2L11_BCL2L11/CSMD1_CSMD1/TSC2_TSC2	1 (8.3%)

### 3.5 Enrichment analysis of GO and KEGG

**Figure 3:**
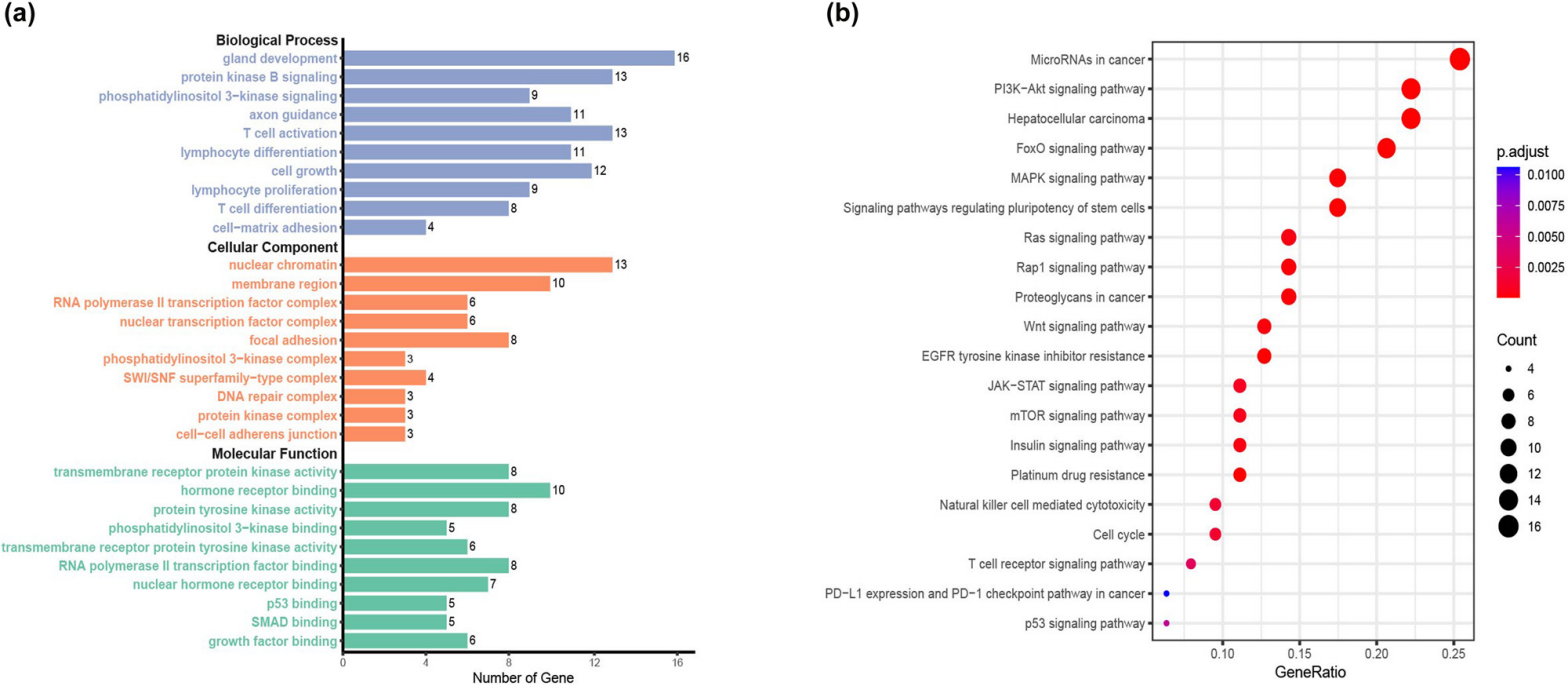
GO and KEGG pathway enrichment analysis of patients with ICCA. (a) GO term enrichment of BP, CC, and MF. (b) Top mutated KEGG pathways in the ICCA.

Functional enrichment analysis was conducted on the enrolled ICCA patients to explore their functions and molecular mechanisms. As shown in Figure 3a, the biological process (BP) of GO was mainly enriched in gland development, protein kinase B signaling, axon guidance, T-cell activation, lymphocyte differentiation, cell growth, lymphocyte proliferation, T-cell differentiation, and cell–matrix adhesion. The molecular function (MF) of GO was mainly enriched in transmembrane receptor protein kinase activity, hormone receptor binding, protein tyrosine kinase activity, phosphatidylinositol 3-kinase binding, transmembrane receptor protein tyrosine kinase activity, RNA polymerase II transcription factor binding, nuclear hormone receptor binding, p53 binding, SMAD binding, and growth factor binding. The cellular component (CC) of GO was mainly enriched in nuclear chromatin, membrane region, RNA polymerase II transcription factor complex, nuclear transcription factor complex, focal adhesion, phosphatidylinositol 3-kinase complex, SWI/SNF superfamily-type complex, DNA repair complex, protein kinase complex, and cell–cell adheren junction. As shown in Figure 3b, the KEGG data revealed that actionable alterations were enriched in various pathways, including the FoxO signaling pathway, hepatocellular carcinoma, microRNAs in cancer, signaling pathways regulating pluripotency of stem cells, *EGFR* tyrosine kinase inhibitor resistance, MAPK signaling pathway, proteoglycans in cancer, Rap1 signaling pathway, Wnt signaling pathway, Ras signaling pathway, natural killer cell-mediated cytotoxicity, T-cell receptor signaling pathway, p53 signaling pathway and PD-L1 expression and PD-1 checkpoint pathway in cancer, etc. In addition, we used the data of the TCGA dataset for enrichment analysis, and the results showed that the BP of GO was mainly enriched in gland development, T-cell differentiation, and neuron projection guidance. The MF of GO was mainly enriched in protein serine/threonine/tyrosine kinase activity, protein tyrosine kinase activity, transmembrane receptor protein kinase activity, and metal ion transmembrane transporter activity. The CC of GO was mainly enriched in cell leading edge, SWI/SNF superfamily-type complex, and cell–cell junction (Figure S2a). KEGG analysis revealed that actionable alterations were enriched in human papillomavirus infection, MAPK signaling pathway, and focal adhesion (Figure S2b). Most of the results of TCGA enrichment analysis are similar to ours, which further validates the conclusions of our study.

Unfortunately, as shown in Table 3, no significant differences were found between KEGG pathways and the prognosis of PFS and OS in ICCA patients. For example, although the pathways of PD-L1 expression and PD-1 checkpoint in cancer, T-cell receptor signaling pathway, and natural killer cell-mediated cytotoxicity seemed to be associated with shorter OS, the *p* values were higher than 0.05 (0.08, 0.07, and 0.07, respectively) (Table 3).

**Table 3:** Associations between pathways and prognosis in ICCA patients

Pathway	*p*-Value	
	PFS	OS
p53 signaling pathway	0.683091	1
PD-L1 expression and PD-1 checkpoint pathway in cancer	0.214193	0.08201
T cell receptor signaling pathway	1	0.066393
Cell cycle	0.683091	1
Natural killer cell mediated cytotoxicity	1	0.066393
Platinum drug resistance	0.619297	0.771926
Insulin signaling pathway	0.567628	0.182287
mTOR signaling pathway	1	0.333949
JAK-STAT signaling pathway	0.396144	0.226256
EGFR tyrosine kinase inhibitor resistance	0.619297	0.333949
Wnt signaling pathway	0.683091	1
Proteoglycans in cancer	0.706082	0.825863
Rap1 signaling pathway	0.619297	0.333949
Ras signaling pathway	0.298698	0.544329
Signaling pathways regulating pluripotency of stem cells	0.298698	0.544329
MAPK signaling pathway	0.362355	0.333949
FoxO signaling pathway	0.665006	0.544329
Hepatocellular carcinoma	0.100348	1
PI3K-Akt signaling pathway	0.706082	0.825863
MicroRNAs in cancer	0.706082	0.825863

### 3.6 Associations between DDR pathway, tumor burden, and prognosis

**Figure 4:**
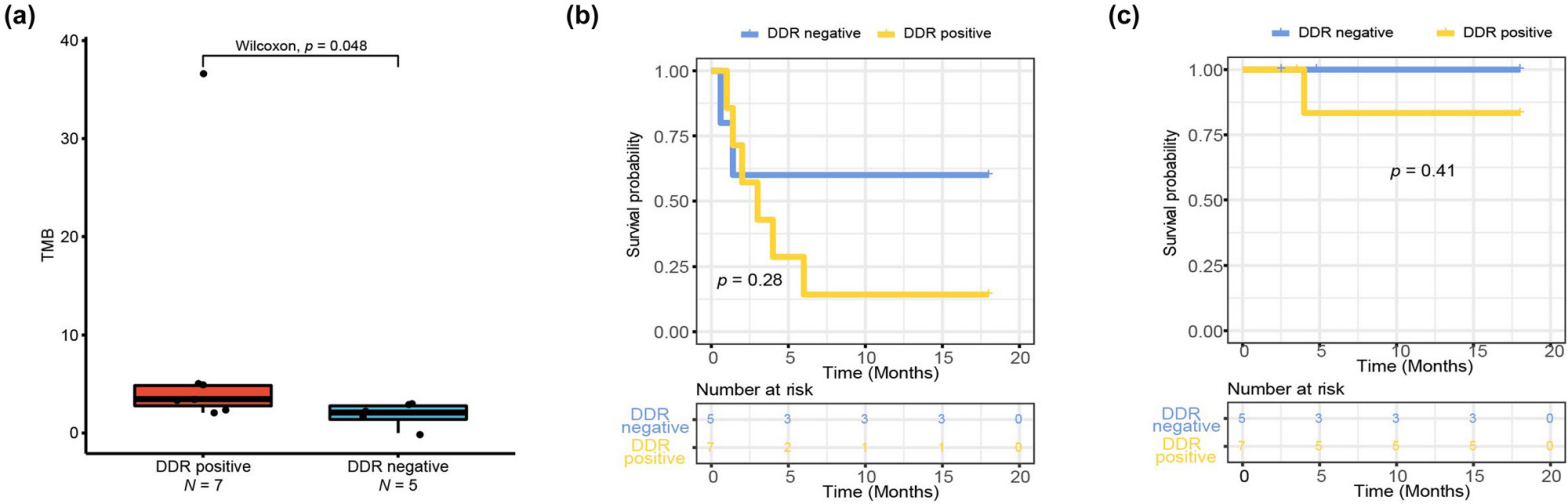
DNA damage response (DRR) pathway alteration status in patients with ICCA. (a) Patients with positive DRR had a higher TMB compared with patients with negative DRR. (b) PFS and (c) OS of all patients stratified by DDR positive versus negative.

Seven tumor samples out of 12 ICCA patients (58.3%) were classified as DDR positive, while the remaining 5 (41.7%) were defined as DDR negative (Table 4). All the five patients with mutations in DDR genes were male and had metastasis. Importantly, we found that DDR-positive patients had a higher frequency of driver gene mutations and had a significant increase in TMB (Figure 4a). Among the eight ICCA cases treated with ICI, those with DDR positive exhibited high TMB, especially in DDR-positive patients treated with first-line of ICI (Figure S3a).

**Table 4:** Associations between clinical impact factors and DDR/HRR mutations

	Group	DDR/HRR positive	DDR/HRR negative	*p* value
**Total patients (** ***N*** **= 12)**		7	5	
Age	>64 years	4	3	0.5581
	≤64 years	3	2	
Gender	Male	5	3	1
	Female	2	2	
Metastasis	Yes	5	3	1
	No	2	2	

**Driver mutations (** ***N*** **= 100)**		86	14	
	*TP53*/*KRAS*	6	2	0.3106
	Others	80	12	

To assess the association between DDR alterations and clinical outcomes, we compared survival rates of patients with DDR-negative and DDR-positive; however, no significant clinical association was found (*p* > 0.05; Figure 4b and c). In addition, we evaluated the predictive role of DDR in patients with ICI treatment, and the results showed that there were no significant differences between the status of DDR and survival (*p* > 0.05; Figure S3b and c). Similarly, homologous recombination repair (HRR) pathway alteration status in patients with ICCA had an identical mutated pattern to those with DDR patients, so we have combined these two parts of data into one part, as shown in Table 4.

## 4 Discussion

In this study, we used a targeted NGS panel to analyze comprehensive genomic profiling on tumor tissue specimens from 12 Chinese ICCA patients. The most frequently mutated genes were identified, among which *TP53* and *KRAS* were the two most frequently mutated genes. The CNV distribution and pathways enrichment of all the mutated genes were also analyzed. Then, we performed survival analysis including the associations between patients with *TP53* and/or *KRAS* mutations and survival, TMB and survival, and treatments and survival. We found that there were significant differences in patients with *TP53* and/or *KRAS* mutations and PFS and in patients with TMB high and PFS. Furthermore, we investigated the survival rates of both PFS and OS in ICCA patients with *KRAS* and/or *TP53* mutations who received ICI treatment. However, no significant differences were observed. Importantly, we found that DDR/HRR-positive patients had a higher frequency of driver gene mutations and had a significant increase in TMB, especially in DDR/HRR-positive patients treated with first-line of ICI. To further validate our conclusions, we performed prognosis and pathway enrichment analyses using data from the ICGC and TCGA datasets, and the results were similar to ours.

Due to its high invasiveness and heterogeneity, ICCA is frequently diagnosed late with an unsatisfied prognosis [1,2,8,31]. With the development of next-sequencing generation technologies and bioinformatics, molecular profiling tends to be a promising biomarker that could be translated into clinical practice of ICCA patients. Personalized therapy based on these biomarkers could potentially improve patients' survival rates [13,16,31,32,33,34,35]. Previous studies have revealed that common genomic alterations in biliary tract cancers, including *TP53*, *KRAS*, *SMAD4*, *ARID1A*, *CDKN2A*, *IDH1,* and *PIK3CA* mutations [3,15,16,19,20,21,36,37]. In a study of the Chinese population, the results showed that the most commonly mutated genes were *TP53* (34%), *KRAS* (25%), and *ARID1A* (17%) [16]. Chen et al.’s study demonstrated that *TP53*, *KRAS,* and *ARID1A* were the top frequently mutated driver genes [36]. Another study of Chinese patients also showed similar results [17]. Our results certainly showed consistent with those in these studies.

*KRAS* and *TP53* mutations have been identified as major driver oncogenes in various cancer tissues, including biliary tract cancer, lung cancer, pancreatic ductal carcinoma, colorectal carcinoma, and laryngeal cancer [3,16,17,19,38,39,40,41,42]. Genetically engineered mouse models have shown that the oncogenic *KRAS* effectors *CREB1* can interact with mutant *p53* to activate a transcriptional network, which promotes metastasis [43]. According to previous studies, these two genes commonly mutated in biliary tract cancer with crucial roles in immunotherapy response and are associated with unfavorable prognosis [13,19,34,36,44]. As expected, ICCA patients with *TP53* and/or *KRAS* mutations had a worse survival rate of PFS than those with wild-type genotypes in this study. However, due to the limited number of ICCA cases, no significant association was found between *TP53* and/or *KRAS* mutations and TMB high. We must acknowledge this limitation in our study; nevertheless, it was still observed that high TMB was associated with worse PFS in ICCA patients than those with low TMB. These clinical outcomes indicated that *TP53* and/or *KRAS* mutations can be used as predictors of poor prognosis in ICCA patients.

It has been observed that co-mutations in *KRAS* and *TP53* result in immune signatures that are enriched in innate immune cells and exclude CD8+ T-cells. Additionally, the transcriptomes altered by *KRAS* and *TP53* co-mutations interact with *TP63*-defined squamous trans-differentiation and myeloid cell migration into the tumor microenvironment [45]. These alterations may be linked to deficient DNA damage repair (DDR), leading to an increased mutation load and immunogenicity of cancer cells [46]. Targeting the DNA damage response (DDR) pathway is a crucial strategy for cancer treatment, and the efficacy of DDR-targeted drugs has been assessed in various types of cancer [47]. Studies have shown that the presence of DDR mutations is significantly correlated with a higher TMB in cholangiocarcinoma, and patients with *BRCA2* germline truncation mutations show an objective response [48]. In patients with biliary tract cancer who have received chemotherapies, those with germline or somatic mutations in DDR genes had significantly longer PFS and OS [49]. Frequent alterations in both copies of certain genes associated with HRR are more commonly found in breast, ovarian, pancreatic, and prostate cancer, which is important for the development of the next generation of clinical trials for DNA repair-targeting drugs [50]. Tumors with HRR deficiency are also highly responsive to other types of DNA-damaging treatments, such as platinum-based chemotherapies [51]. Our data showed that DDR deficiency and HRR alteration were 58.3% (7/12) in ICCA samples, which is similar to previous findings [46,48], and DDR mutation was associated with high TMB in ICCA samples.

We also identified *ETV6* amplification, *CDKN2A* deletions, and *MEF2B*_*MEF2B* fusion in ICCA tissues. Notably, the amplification of *ETV6* has been identified as a potential oncogene of leukemia [52]. *CDKN2A* deletions have been shown to inhibit T-cell infiltration by modulating MAPK and NF-κB signaling pathways in a cell cycle-dependent manner [53]. Additionally, B-cell acute lymphoblastic leukemia patients with *CDKN2A*/*2B* deletions exhibited poor 2 year OS and relapse-free survival rates [54]. *MEF2BNB*-*MEF2B* fusion was found to be prevalent in various cancers such as astrocytoma, cancer of unknown primary, NOS (not otherwise specified), esophagogastric carcinoma, meningioma, and mycosis fungoides in AACR Project GENIE cases [55].

## 5 Conclusions

Our result identified frequent common mutations of *TP53* and *KRAS* among ICCA patients, which are commonly observed in this type of cancer. More importantly, patients with *TP53* and/or *KRAS* alterations were significantly associated with poor prognosis. Meanwhile, TMB high was found to be correlated with DDR mutation genes and HRR mutation genes. These results indicated that certain genomic alterations contribute to the clinical heterogeneity of ICCA. However, further research with larger sample sizes is required for a more comprehensive and deeper understanding of the molecular mechanisms underlying the development of this cancer.

## Supplementary Material

Supplementary material

## References

[1] Lee YT, Wang JJ, Luu M, Noureddin M, Nissen NN, Patel TC, et al. Comparison of clinical features and outcomes between intrahepatic cholangiocarcinoma and hepatocellular carcinoma in the united states. Hepatology. 2021;74(5):2622–32. 10.1002/hep.32007 34114675

[2] Javle M, Lee S, Azad NS, Borad MJ, Kate Kelley R, Sivaraman S, et al. Temporal changes in cholangiocarcinoma incidence and mortality in the United States from 2001 to 2017. Oncologist. 2022;27(10):874–83. 10.1093/oncolo/oyac150 PMC9526482 35972334

[3] Guo L, Zhou F, Liu H, Kou X, Zhang H, Chen X, et al. Genomic mutation characteristics and prognosis of biliary tract cancer. Am J Transl Res. 2022;14(7):4990–5002. PMC9360853 35958441

[4] Brindley PJ, Bachini M, Ilyas SI, Khan SA, Loukas A, Sirica AE, et al. Cholangiocarcinoma. Nat Rev Dis Primers. 2021;7(1):65. 10.1038/s41572-021-00300-2 PMC9246479 34504109

[5] Kam AE, Masood A, Shroff RT. Current and emerging therapies for advanced biliary tract cancers. Lancet Gastroenterol Hepatol. 2021;6(11):956–69. 10.1016/S2468-1253(21)00171-0 34626563

[6] Florio AA, Ferlay J, Znaor A, Ruggieri D, Alvarez CS, Laversanne M, et al. Global trends in intrahepatic and extrahepatic cholangiocarcinoma incidence from 1993 to 2012. Cancer. 2020;126(11):2666–78. 10.1002/cncr.32803 PMC7323858 32129902

[7] Xing H, Tan B, Yang C, Zhang M. Incidence trend and competing risk analysis of patients with intrahepatic cholangiocarcinoma: A population-based study. Front Med (Lausanne). 2022;9:846276. 10.3389/fmed.2022.846276 PMC9005886 35433765

[8] Yao J, Liang X, Liu Y, Li S, Zheng M. Trends in incidence and prognostic factors of two subtypes of primary liver cancers: A surveillance, epidemiology, and end results-based population study. Cancer Control. 2022;29:10732748211051548. 10.1177/10732748211051548 PMC8841928 35147456

[9] Banales JM, Marin JJG, Lamarca A, Rodrigues PM, Khan SA, Roberts LR, et al. Cholangiocarcinoma 2020: the next horizon in mechanisms and management. Nat Rev Gastroenterol Hepatol. 2020;17(9):557–88. 10.1038/s41575-020-0310-z PMC7447603 32606456

[10] Zeng H, Chen W, Zheng R, Zhang S, Ji JS, Zou X, et al. Changing cancer survival in China during 2003-15: a pooled analysis of 17 population-based cancer registries. Lancet Glob Health. 2018;6(5):e555–67. 10.1016/S2214-109X(18)30127-X 29653628

[11] Lin J, Dai Y, Sang C, Song G, Xiang B, Zhang M, et al. Multimodule characterization of immune subgroups in intrahepatic cholangiocarcinoma reveals distinct therapeutic vulnerabilities. J Immunother Cancer. 2022;10(7):e004892. 10.1136/jitc-2022-004892 PMC9310257 35863823

[12] Biller LH, Schrag D. Diagnosis and treatment of metastatic colorectal cancer: A review. JAMA. 2021;325(7):669–85. 10.1001/jama.2021.0106 33591350

[13] Sarantis P, Tzanetatou ED, Ioakeimidou E, Vallilas C, Androutsakos T, Damaskos C, et al. Cholangiocarcinoma: the role of genetic and epigenetic factors; current and prospective treatment with checkpoint inhibitors and immunotherapy. Am J Transl Res. 2021;13(12):13246–60. PMC8748131 35035673

[14] Rosati G, Aprile G, Colombo A, Cordio S, Giampaglia M, Cappetta A, et al. Colorectal cancer heterogeneity and the impact on precision medicine and therapy efficacy. Biomedicines. 2022;10(5):1035. 10.3390/biomedicines10051035 PMC9138254 35625772

[15] Wardell CP, Fujita M, Yamada T, Simbolo M, Fassan M, Karlic R, et al. Genomic characterization of biliary tract cancers identifies driver genes and predisposing mutations. J Hepatol. 2018;68(5):959–69. 10.1016/j.jhep.2018.01.009 29360550

[16] Wang L, Zhu H, Zhao Y, Pan Q, Mao A, Zhu W, et al. Comprehensive molecular profiling of intrahepatic cholangiocarcinoma in the Chinese population and therapeutic experience. J Transl Med. 2020;18(1):273. 10.1186/s12967-020-02437-2 PMC7336472 32631434

[17] Guo C, Liu Z, Yu Y, Chen Y, Liu H, Guo Y, et al. TP53/KRAS Co-mutations create divergent prognosis signatures in intrahepatic cholangiocarcinoma. Front Genet. 2022;13:844800. 10.3389/fgene.2022.844800 PMC8990229 35401671

[18] Hill MA, Alexander WB, Guo B, Kato Y, Patra K, O’Dell MR, et al. Kras and Tp53 mutations cause cholangiocyte- and hepatocyte-derived cholangiocarcinoma. Cancer Res. 2018;78(16):4445–51. 10.1158/0008-5472.CAN-17-1123 PMC6097629 29871934

[19] Wang XY, Zhu WW, Wang Z, Huang JB, Wang SH, Bai FM, et al. Driver mutations of intrahepatic cholangiocarcinoma shape clinically relevant genomic clusters with distinct molecular features and therapeutic vulnerabilities. Theranostics. 2022;12(1):260–76. 10.7150/thno.63417 PMC8690927 34987644

[20] Yu H, Xu Y, Gao W, Li M, He J, Deng X, et al. Comprehensive germline and somatic genomic profiles of Chinese patients with biliary tract cancer. Front Oncol. 2022;12:930611. 10.3389/fonc.2022.930611 PMC9441936 36072793

[21] Takada K, Kubo T, Kikuchi J, Yoshida M, Murota A, Arihara Y, et al. Effect of comprehensive cancer genomic profiling on therapeutic strategies and clinical outcomes in patients with advanced biliary tract cancer: A prospective multicenter study. Front Oncol. 2022;12:988527. 10.3389/fonc.2022.988527 PMC9478541 36119486

[22] Roosan MR, Mambetsariev I, Pharaon R, Fricke J, Baroz AR, Chao J, et al. Evaluation of somatic mutations in solid metastatic pan-cancer patients. Cancers (Basel). 2021;13(11):2776. 10.3390/cancers13112776 PMC8199748 34204917

[23] Zhang Y, Yao Y, Xu Y, Li L, Gong Y, Zhang K, et al. Pan-cancer circulating tumor DNA detection in over 10,000 Chinese patients. Nat Commun. 2021;12(1):11. 10.1038/s41467-020-20162-8 PMC7782482 33397889

[24] Huang H, Deng T, Guo Y, Chen H, Cui X, Duan J, et al. Gene mutational clusters in the tumors of colorectal cancer patients with a family history of cancer. Front Oncol. 2022;12:814397. 10.3389/fonc.2022.814397 PMC9266985 35814400

[25] Kaubryte J, Lai AG. Pan-cancer prognostic genetic mutations and clinicopathological factors associated with survival outcomes: a systematic review. NPJ Precis Oncol. 2022;6(1):27. 10.1038/s41698-022-00269-5 PMC9021198 35444210

[26] Zhang M, Wu J, Zhong W, Zhao Z, Guo W. Comparative study on the mutation spectrum of tissue DNA and blood ctDNA in patients with non-small cell lung cancer. Transl Cancer Res. 2022;11(5):1245–54. 10.21037/tcr-22-970 PMC9189250 35706796

[27] Chalmers ZR, Connelly CF, Fabrizio D, Gay L, Ali SM, Ennis R, et al. Analysis of 100,000 human cancer genomes reveals the landscape of tumor mutational burden. Genome Med. 2017;9(1):34. 10.1186/s13073-017-0424-2 PMC5395719 28420421

[28] Cancer Genome Atlas Research Network. Electronic address edsc, Cancer Genome Atlas Research N. Comprehensive and integrated genomic characterization of adult soft tissue sarcomas. Cell. 2017;171(4):950–65 e28. 10.1016/j.cell.2017.10.014 PMC5693358 29100075

[29] Newman AM, Bratman SV, Stehr H, Lee LJ, Liu CL, Diehn M, et al. FACTERA: a practical method for the discovery of genomic rearrangements at breakpoint resolution. Bioinformatics. 2014;30(23):3390–3. 10.1093/bioinformatics/btu549 PMC4296148 25143292

[30] Liu J, Han F, Ding J, Liang X, Liu J, Huang D, et al. Identification of multiple hub genes and pathways in hepatocellular carcinoma: A bioinformatics analysis. Biomed Res Int. 2021;2021:8849415. 10.1155/2021/8849415 PMC8292096 34337056

[31] Tomczak A, Springfeld C, Dill MT, Chang DH, Kazdal D, Wagner U, et al. Precision oncology for intrahepatic cholangiocarcinoma in clinical practice. Br J Cancer. 2022;127(9):1701–8. 10.1038/s41416-022-01932-1 PMC9390961 35986087

[32] Levillain H, Duran Derijckere I, Ameye L, Guiot T, Braat A, Meyer C, et al. Personalised radioembolization improves outcomes in refractory intra-hepatic cholangiocarcinoma: a multicenter study. Eur J Nucl Med Mol Imaging. 2019;46(11):2270–9. 10.1007/s00259-019-04427-z 31324943

[33] Wu Q, Zhen Y, Shi L, Vu P, Greninger P, Adil R, et al. EGFR inhibition potentiates FGFR inhibitor therapy and overcomes resistance in FGFR2 fusion-positive cholangiocarcinoma. Cancer Discov. 2022;12(5):1378–95. 10.1158/2159-8290.CD-21-1168 PMC9064956 35420673

[34] Yoon JG, Kim MH, Jang M, Kim H, Hwang HK, Kang CM, et al. Molecular characterization of biliary tract cancer predicts chemotherapy and programmed death 1/programmed death-ligand 1 blockade responses. Hepatology. 2021;74(4):1914–31. 10.1002/hep.31862 33884649

[35] Zhang Z, Zhang W, Wang H, Hu B, Wang Z, Lu S. Successful treatment of advanced intrahepatic cholangiocarcinoma with a high tumor mutational burden and PD-L1 expression by PD-1 blockade combined with tyrosine kinase inhibitors: A case report. Front Immunol. 2021;12:744571. 10.3389/fimmu.2021.744571 PMC8484748 34603331

[36] Chen X, Wang D, Liu J, Qiu J, Zhou J, Ying J, et al. Genomic alterations in biliary tract cancer predict prognosis and immunotherapy outcomes. J Immunother Cancer. 2021;9(11):e003214. 10.1136/jitc-2021-003214 PMC8603283 34795005

[37] Lin J, Cao Y, Yang X, Li G, Shi Y, Wang D, et al. Mutational spectrum and precision oncology for biliary tract carcinoma. Theranostics. 2021;11(10):4585–98. 10.7150/thno.56539 PMC7978308 33754015

[38] Hu C, Zhao L, Liu W, Fan S, Liu J, Liu Y, et al. Genomic profiles and their associations with TMB, PD-L1 expression, and immune cell infiltration landscapes in synchronous multiple primary lung cancers. J Immunother Cancer. 2021;9(12):e003773. 10.1136/jitc-2021-003773 PMC8663088 34887263

[39] Ecker BL, Court CM, Janssen QP, Tao AJ, D’Angelica MI, Drebin JA, et al. Alterations in somatic driver genes are associated with response to neoadjuvant FOLFIRINOX in patients with localized pancreatic ductal adenocarcinoma. J Am Coll Surg. 2022;235(2):342–9. 10.1097/XCS.0000000000000212 PMC9319357 35839413

[40] Jan YH, Tan KT, Chen SJ, Yip TTC, Lu CT, Lam AK. Comprehensive assessment of actionable genomic alterations in primary colorectal carcinoma using targeted next-generation sequencing. Br J Cancer. 2022;127(7):1304–11. 10.1038/s41416-022-01913-4 PMC9519871 35842545

[41] Mezghani N, Yao A, Vasilyeva D, Kaplan N, Shackelford A, Yoon A, et al. Molecular subtypes of head and neck cancer in patients of African ancestry. Clin Cancer Res. 2023;29(5):910–20. 10.1158/1078-0432.CCR-22-2258 PMC9991972 36508165

[42] Insodaite R, Smalinskiene A, Liutkevicius V, Ulozas V, Poceviciute R, Bielevicius A, et al. Associations of polymorphisms localized in the 3′UTR regions of the KRAS, NRAS, MAPK1 genes with laryngeal squamous cell carcinoma. Genes (Basel). 2021;12(11):1679. 10.3390/genes12111679 PMC8625477 34828284

[43] Kim MP, Li X, Deng J, Zhang Y, Dai B, Allton KL, et al. Oncogenic KRAS recruits an expansive transcriptional network through mutant p53 to drive pancreatic cancer metastasis. Cancer Discov. 2021;11(8):2094–111. 10.1158/2159-8290.CD-20-1228 PMC8338884 33839689

[44] Kwack WG, Shin SY, Lee SH. Primary resistance to immune checkpoint blockade in an STK11/TP53/KRAS-mutant lung adenocarcinoma with high PD-L1 expression. Onco Targets Ther. 2020;13:8901–5. 10.2147/OTT.S272013 PMC7494390 32982282

[45] Datta J, Bianchi A, De Castro Silva I, Deshpande NU, Cao LL, Mehra S, et al. Distinct mechanisms of innate and adaptive immune regulation underlie poor oncologic outcomes associated with KRAS-TP53 co-alteration in pancreatic cancer. Oncogene. 2022;41(28):3640–54. 10.1038/s41388-022-02368-w 35701533

[46] Gu M, Xu T, Chang P. KRAS/LKB1 and KRAS/TP53 co-mutations create divergent immune signatures in lung adenocarcinomas. Ther Adv Med Oncol. 2021;13:17588359211006950. 10.1177/17588359211006950 PMC8072935 33995590

[47] Gottifredi V. Targeting DNA damage response kinases in cancer therapy. Mutat Res. 2020;821:111725. 10.1016/j.mrfmmm.2020.111725 33157476

[48] Lin J, Shi J, Guo H, Yang X, Jiang Y, Long J, et al. Alterations in DNA damage repair genes in primary liver cancer. Clin Cancer Res. 2019;25(15):4701–11. 10.1158/1078-0432.CCR-19-0127 31068370

[49] Chae H, Kim D, Yoo C, Kim KP, Jeong JH, Chang HM, et al. Therapeutic relevance of targeted sequencing in management of patients with advanced biliary tract cancer: DNA damage repair gene mutations as a predictive biomarker. Eur J Cancer. 2019;120:31–9. 10.1016/j.ejca.2019.07.022 31476489

[50] Westphalen CB, Fine AD, André F, Ganesan S, Heinemann V, Rouleau E, et al. Pan-cancer analysis of homologous recombination repair-associated gene alterations and genome-wide loss-of-heterozygosity score. Clin Cancer Res. 2022;28(7):1412–21. 10.1158/1078-0432.CCR-21-2096 PMC8982267 34740923

[51] O’Reilly EM, Lee JW, Zalupski M, Capanu M, Park J, Golan T, et al. Randomized, multicenter, phase II trial of gemcitabine and cisplatin with or without veliparib in patients with pancreas adenocarcinoma and a germline BRCA/PALB2 mutation. J Clin Oncol. 2020;38(13):1378–88. 10.1200/JCO.19.02931 PMC7193749 31976786

[52] Chae H, Kim M, Lim J, Kim Y, Han K, Lee S. B lymphoblastic leukemia with ETV6 amplification. Cancer Genet Cytogenet. 2010;203(2):284–7. 10.1016/j.cancergencyto.2010.08.004 21156245

[53] Zhu Z, Song H, Xu J. CDKN2A deletion in melanoma excludes T cell infiltration by repressing chemokine expression in a cell cycle-dependent manner. Front Oncol. 2021;11:641077. 10.3389/fonc.2021.641077 PMC8027313 33842347

[54] Fang Q, Yuan T, Li Y, Feng J, Gong X, Li Q, et al. Prognostic significance of copy number alterations detected by multi-link probe amplification of multiple genes in adult acute lymphoblastic leukemia. Oncol Lett. 2018;15(4):5359–67. 10.3892/ol.2018.7985 PMC5840682 29552179

[55] Consortium APG. AACR project GENIE: Powering precision medicine through an international consortium. Cancer Discov. 2017;7(8):818–31. 10.1158/2159-8290.CD-17-0151 PMC5611790 28572459

