# Supplementary material for: Genetic alterations of KRAS and TP53 in intrahepatic cholangiocarcinoma associated with poor prognosis
Source: Open Life Sci. 2023 Jul 17;18(1):20220652. doi: 10.1515/biol-2022-0652 (PMC10358752; doi:10.1515/biol-2022-0652)
Supplement: Supplementary material [file biol-2022-0652-sm.pdf]

Supplementary material

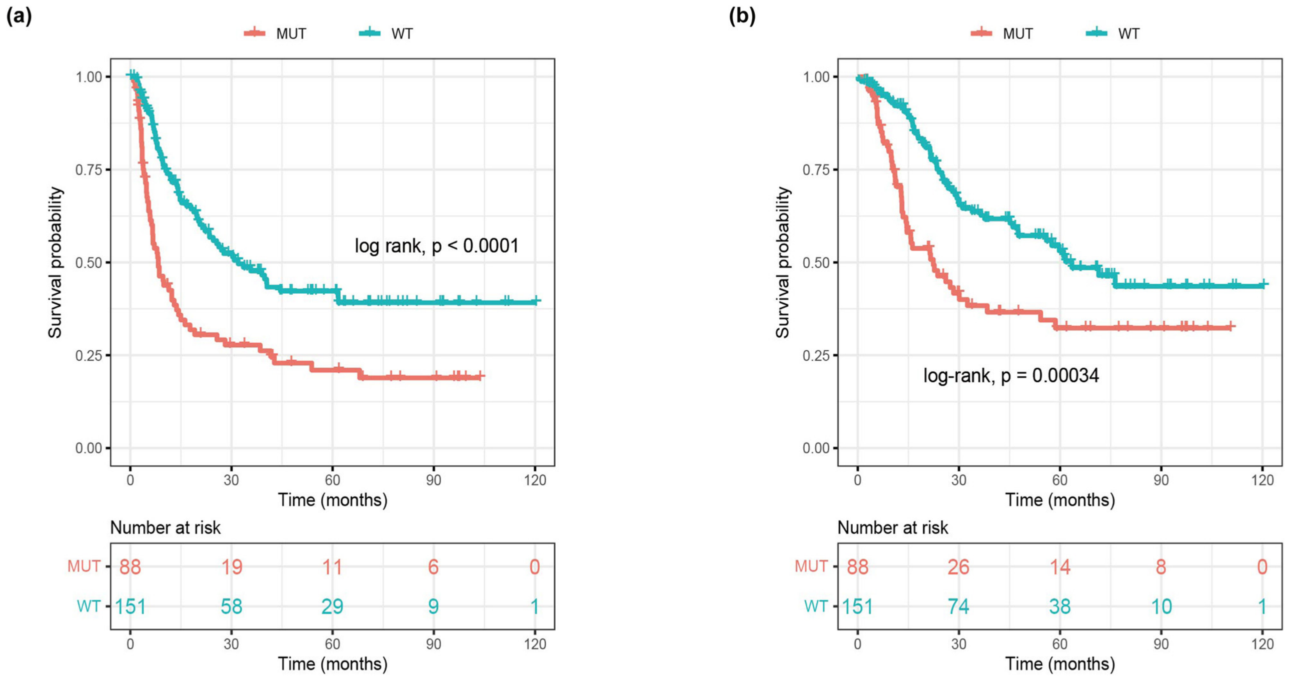

Figure S1: Prognosis of ICCA with KRAS and/or TP53 from ICGC database. (a) Progressive free survival (PFS) and (b) overall survival (OS) of all patients stratified by TP53 and or KRAS mutation versus wild type (MUT vs WT).

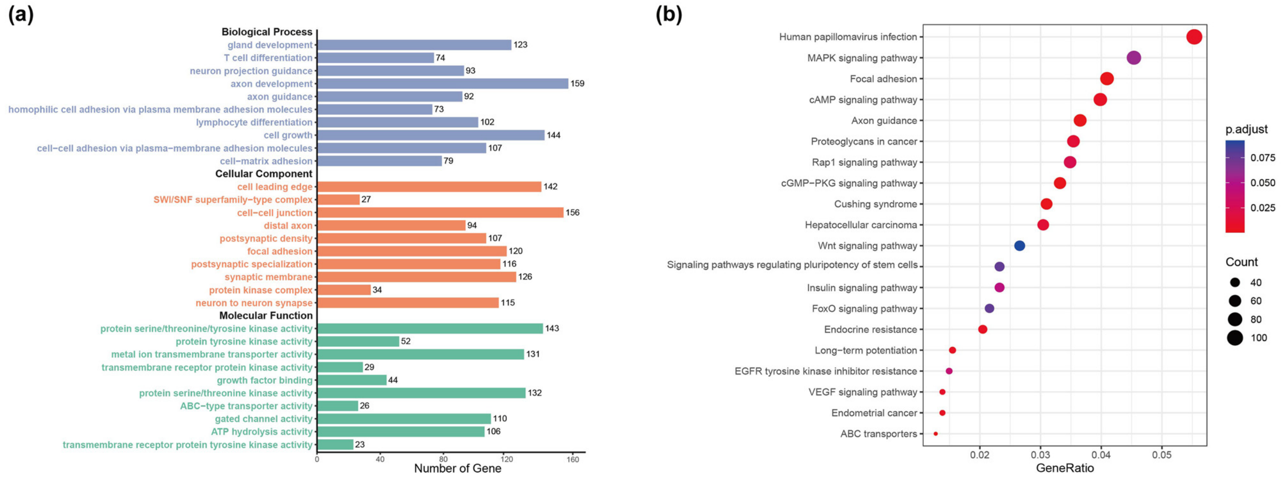

Figure S2: GO and KEGG pathway enrichment analysis of patients with ICCA from TCGA database. (a) GO term enrichment of biological process, cellular component, and molecular function. (b) Top mutated KEGG pathways in the ICCA.

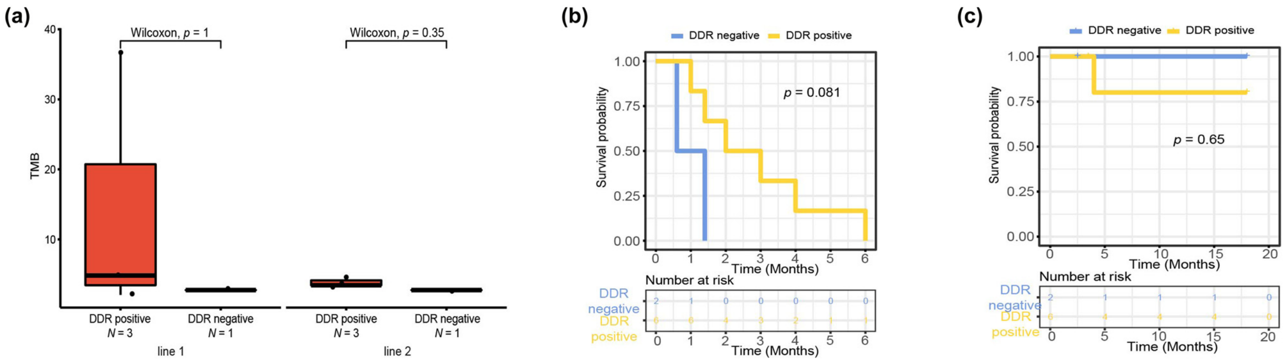

Figure S3: DRR pathway alteration status in ICCA patients treated by immune checkpoint inhibitor (ICI). (a) Patients with positive DRR had a higher TMB compared with patients with negative DRR both in line 1 and 2 of ICI treatment. (b) (PFS and (c) OS of all patients stratified by DDR positive versus negative was shown in patients treated with ICI.

Table S1: Prognosis of *KRAS* & *TP53* mutations in ICCA patients treated with ICI

	Patient number (*N* = 12)	Median PFS (Months)	Median OS (Months)	*P* value (PFS)	*P* value (OS)
KRAS & *TP53* mutations	5	1.4	4	0.76	0.17
Wild type	3	3	18		

Table S2: Associations between patients with *KRAS* & *TP53* mutation and TMB, and clinical characteristics

Feature	Group	*P* value (*KRAS* & *TP53* mutations vs wild type)	*P* value (TMB high vs TMB low)
Age	≥64 years	0.5581	1
	<64 years		
TNM stage	I–II	0.2929	1
	III–IV		
Metastasis	Yes	0.5758	0.5758
	No		
Origin site	Left	0.5227	0.2045
	Right		
Tumor size	≥180 cm^3	1	0.5671
	<180 cm^3		
PD-L1	Positive	1	0.4444
	Negative		
OS	≥18 months	0.2222	0.5758
	<18 months		
TMB	≥2.76 mutations/Mb	0.2929	—
	<2.76 mutations/Mb		

Table S3: Univariate Cox model analysis between the top 20 mutated genes and overall survival

Genes	Hazard ratio	*P* value
ACVR1B	−19.1681	0.999649
APC	−19.36	0.999517
ARID1A	23.04216	0.999635
ARID2	−19.36	0.999517
ATM	−19.36	0.999517
CTNNB1	−19.36	0.999517
DNMT1	−19.1681	0.999649
FGFR2	−19.1681	0.999649
GNAS	−19.1681	0.999649
KRAS	29.00064	0.999965
PBRM1	−19.36	0.999517
PIK3CA	−19.36	0.999517
RASA1	−19.1681	0.999649
RBM10	−19.36	0.999517
RUNX1	23.04216	0.999635
SMAD4	29.00064	0.999965
SUFU	−19.1681	0.999649
TP53	22.46062	0.999637
